# Betaine and Secondary Events in an Acute Coronary Syndrome Cohort

**DOI:** 10.1371/journal.pone.0037883

**Published:** 2012-05-23

**Authors:** Michael Lever, Peter M. George, Jane L. Elmslie, Wendy Atkinson, Sandy Slow, Sarah L. Molyneux, Richard W. Troughton, A. Mark Richards, Christopher M. Frampton, Stephen T. Chambers

**Affiliations:** 1 Clinical Biochemistry Unit, Canterbury Health Laboratories, Christchurch, New Zealand; 2 Department of Pathology, University of Otago (Christchurch), Christchurch, New Zealand; 3 The Christchurch Cardioendocrine Research Group, Department of Medicine, University of Otago (Christchurch), Christchurch, New Zealand; Virginia Commonwealth University, United States of America

## Abstract

**Background:**

Betaine insufficiency is associated with unfavourable vascular risk profiles in metabolic syndrome patients. We investigated associations between betaine insufficiency and secondary events in acute coronary syndrome patients.

**Methods:**

Plasma (531) and urine (415) samples were collected four months after discharge following an acute coronary event. Death (34), secondary acute myocardial infarction (MI) (70) and hospital admission for heart failure (45) events were recorded over a median follow-up of 832 days.

**Principal Findings:**

The highest and lowest quintiles of urinary betaine excretion associated with risk of heart failure (*p* = 0.0046, *p* = 0.013 compared with middle 60%) but not with subsequent acute MI. The lowest quintile of plasma betaine was associated with subsequent acute MI (*p* = 0.014), and the top quintile plasma betaine with heart failure (*p* = 0.043), especially in patients with diabetes (*p*<0.001). Top quintile plasma concentrations of dimethylglycine (betaine metabolite) and top quintile plasma homocysteine both associated with all three outcomes, acute MI (*p* = 0.004, <0.001), heart failure (*p* = 0.027, *p*<0.001) and survival (*p*<0.001, *p*<0.001). High homocysteine was associated with high or low betaine excretion in >60% of these subjects (*p* = 0.017). Median NT-proBNP concentrations were lowest in the middle quintile of plasma betaine concentration (*p* = 0.002).

**Conclusions:**

Betaine insufficiency indicates increased risk of secondary heart failure and acute MI. Its association with elevated homocysteine may partly explain the disappointing results of folate supplementation. In some patients, especially with diabetes, elevated plasma betaine also indicates increased risk.

## Introduction

**Figure 1:**
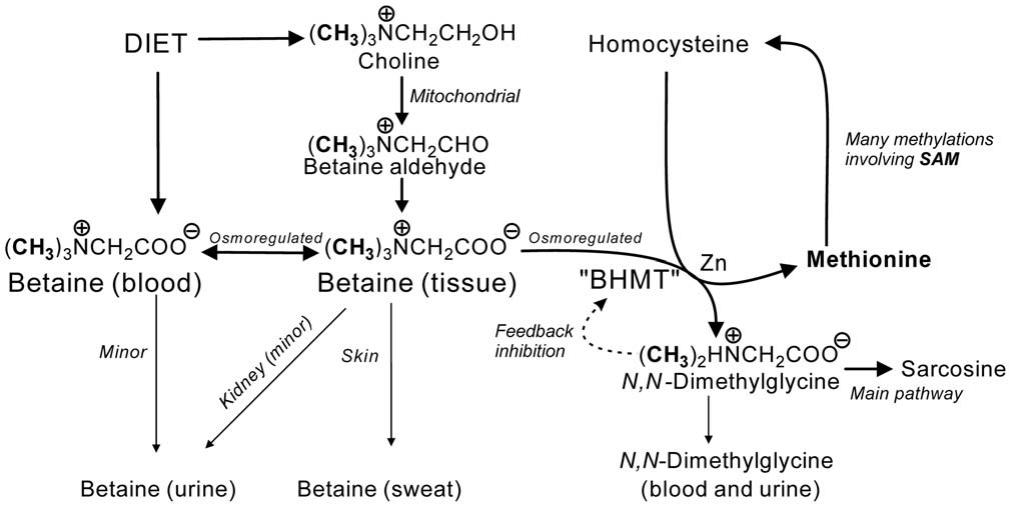
Betaine metabolism. Metabolic pathways involving betaine. BHMT: betaine homocysteine methyltransferase, a zinc metalloenzyme abundant in human liver and kidney tissues. SAM: *S*-adenosylmethionine, ubiquitous methyl-group donor in human metabolism.

Betaine (*N,N,N*-trimethylglycine) is an essential osmolyte and methyl group donor [1]–[4] that also affects lipid partitioning [5]. Its metabolism (**Figure 1**) links several metabolites that play an important role in the health of humans and other mammals, including choline (an important source of betaine), and homocysteine and methionine which are involved in its catabolism. Cross-sectional data [6]–[8] hint that betaine insufficiency may be associated with vascular disease, especially in subjects with the metabolic syndrome [3], but the evidence is circumstantial. Low plasma betaine is common in subjects with an unfavourable vascular risk profile [7], [8], but plasma betaine is only modestly correlated with tissue betaine [9]; because of its role as an osmolyte, betaine concentrations are much higher in most tissues than in blood [9]. Normally minimal amounts are lost in the urine, even after a substantial betaine load [10]–[11], showing that the normal pathway for elimination is by catabolism. The strong homeostatic control of plasma and urine betaine [12]–[14] is only minimally affected by osmotic changes, despite large changes in tissue betaine concentrations, and there is no correlation between plasma betaine concentrations and urinary betaine excretions. Thus while low plasma betaine could be associated with a tissue betaine insufficiency, the plasma concentration is a limited marker. An insufficiency could be the result of excessive loss or defective metabolism of choline to betaine [3], and these could be exacerbated by poor dietary choices. However, other cross-sectional evidence has also associated elevated plasma betaine with vascular disease [15]. This presumably reflects a different pathology; the plasma betaine concentrations in this study were still well below tissue concentrations and the elevations could reflect (for example) defective retention of intracellular betaine in some tissues.

Betaine insufficiency is therefore hard to detect. The methionine load test may be a test of betaine sufficiency [3], [16] but it is not practicable to screen seriously ill subjects with this test. Nevertheless, some biochemical markers may indicate at least some cases of betaine insufficiency. Unusually low plasma betaine is one. Another is a high urinary betaine loss, which could be expected to cause a deficiency; patients with diabetes or renal failure often have either abnormally high or abnormally low betaine excretion [3], [13]. Subjects with a severe betaine insufficiency for some other reason could also be expected to have unusually low betaine excretion. Raised plasma dimethylglycine [3] indicates an increased catabolism of betaine (a response to homocysteine accumulation), and a reduced supply of betaine is an important cause of elevated fasting plasma homocysteine [2], [3]. If homocysteine is elevated in response to a betaine insufficiency, it will not be corrected by B-vitamin supplementation. This could help to explain why this treatment does not lead to the expected reduction in vascular events [17]–[19]; possibly the elevated homocysteine is a marker of betaine insufficiency in a subset of the study populations, rather than causal.

The aim of the present study was to prospectively relate potential markers of betaine insufficiency to acute MI and heart failure, in a high-risk population with established vascular disease. This population would therefore be expected to be enriched with subjects with an abnormal betaine status at baseline.

## Methods

### Ethics

Study protocols were approved by the Canterbury Ethics Committee. All subjects gave written informed consent. The investigation conforms to the principles outlined in the Declaration of Helsinki and Title 45, US Code of Federal Regulations, Part 46 and with published accepted principles [20].

### Subjects

Subjects in the present betaine sub-study were part of an Acute Coronary Syndrome (ACS) cohort recruited by the Christchurch Cardioendocrine Group, Christchurch Hospital [21]. Eligible subjects were recruited following hospitalization for ACS defined by a history of ischemic chest pain plus one or more of the following: ECG changes (ST segment depression or elevation of at least 0.5 mm, T-wave inversion of at least 3 mm in at least 3 leads, or left bundle branch block), elevated levels of cardiac markers, a history of coronary disease, or age of at least 65 years in patients with diabetes or vascular disease. These are identical to the criteria used in the OPUS-TIMI 16 trial and by De Lemos et al. [22]. Exclusion criteria included severe co-morbidity limiting life expectancy to less than 3 years and inability to provide written informed consent. For the betaine sub-study fasting plasma samples were collected on 531 subjects, and matching urine samples on 415 of these, at the four-month post-discharge follow-up outpatient clinic visit (**Table 1**). The patients were expected to have stabilized by this visit.

**Table 1:** Study population.

	Females	Males
Number	148	383
Median age (total range)**	73 (51–91)	67 (55–93)
Follow-up time/time to death (median, IQ range) days	878 (657–1005)	825 (600–988)
With diabetes†, n(%)	28 (19%)	65 (17%)
Previous MI, n(%)	37 (25%)	133 (35%)
Secondary acute MI, n(%)	16 (11%)	54 (14%)
Heart failure, n(%)	13 (9%)	32 (8%)
Deaths (all-cause), n(%)	7 (5%)	27 (7%)
Left ventricular ejection fraction (median, IQ range) %***	63 (55–68)	58 (50.5–63)
Waist (median, IQ range) cm***	89 (80–97)	97 (90–103)
BMI (median, IQ range) kg/m^2^	27.0 (22.7–31.6)	26.5 (24.5–29.4)
*Biochemical and hematological parameters:*		
Hemoglobin (median, IQ range) g/L***	132.5 (122–140)	143.5 (136–152)
Plasma creatinine (median, IQ range) µmol/L***	80 (70–95)	100 (90–112)
Plasma urea (median, IQ range) mmol/L*	6.1 (4.9–7.8)	6.6 (5.5–8.5)
Plasma homocysteine (median, IQ range) µmol/L	12.5 (10.2–15.6)	12.6 (10.6–16.3)
Plasma NT-proBNP (median, IQ range) pmol/L	87.0 (45.9–178)	79.9 (34.7–168)
Plasma betaine (median, IQ range) µmol/L***	38.9 (30.8–46.0)	44.5 (35.0–57.5)
Plasma DMG (median, IQ range) µmol/L*	3.4 (2.2–4.8)	3.8 (2.7–5.2)
Urine betaine/creatinine (median, IQ range) mmol/mole cr	8.2 (4.4–17.7)	9.2 (5.9–17.6)
Urine DMG/creatinine (median, IQ range) mmol/mole cr	2.4 (1.4–4.3)	2.9 (1.5–5.9)
*Smoking:*		
Current smokers, n(%)	12 (8%)	20 (5%)
Past smokers, n(%)***	65 (44%)	241 (63%)
Never smoked, n(%)***	71 (48%)	123 (32%)
*Medications:*		
Taking ACE inhibitors, n(%)	76 (51%)	212 (55%)
Taking β-blocker drugs, n(%)	123 (83%)	328 (86%)
Taking statins, n(%)*	118 (80%)	336 (88%)
Taking aspirin, n(%)	138 (93%)	354 (92%)
Taking Clopidogrel, n(%)*	47 (32%)	156 (41%)

cr: creatinine. DMG: *N,N*-dimethylglycine. IQ: interquartile. NT-proBNP: *N*-terminal peptide of B-type natriuretic peptide. Significance of gender differences:

* *p*<0.05;

** *p*<0.01;

*** *p*<0.001.

† Three patients (2 female, 1 male) with Type 1 diabetes, remainder all Type 2.

### Follow up

In the present sub-study, subjects were followed for a median of 832 days (IQ range 621–991 days) from enrolment. Clinical events were determined from recruitment questionnaires, planned follow-up clinic visits for all patients, patient notes, and the New Zealand Health Information Service and hospital Patient Management System databases. Clinical endpoints included all-cause mortality, hospitalization for acute decompensated heart failure [23] and hospitalization for acute MI [24]. Diagnoses at hospitalisation were defined using the International Statistical Classification of Diseases and Health Related Health Problems 10th Revision (ICD-10).

### Laboratory methods

Betaine and *N,N*-dimethylglycine were measured in plasma and urine by HPLC after separation of their 2-naphthacyl derivatives on Merck Aluspher alumina columns [25], [26]. Comparative data on normal subjects has been reviewed elsewhere [3]. Plasma *N*-terminal pro-brain natriuretic peptide (NT-proBNP) was estimated as previously described [27]. Plasma homocysteine was measured by fluorescence polarization on an Abbott IMX Analyzer (Abbott Laboratories USA). Other biochemical measures in plasma and urine were carried out using an Abbott ARCHITECT ci8200 Analyzer (Abbott Laboratories) by standard kit procedures in an International Accreditation New Zealand accredited laboratory.

Excretions were expressed as mmoles betaine or *N,N*-dimethylglycine per mole creatinine, which has been shown to be a robust measure [14].

### Statistical analyses

Statistical analyses were carried out using SigmaPlot for Windows version 11.2 (Systat Software Inc). Significance was taken as p<0.05. Urine betaine excretion may be either abnormally high or low in disease [13], and therefore we divided data for all variables into quintiles (for both urine and plasma) and compared time to event in each of the top and bottom quintiles with the central 60% of the sample population. When significant effects were identified from these comparisons these were further explored by comparisons among all 5 quintiles. Kaplan-Meier cumulative plots, with death (all-cause), secondary acute MI or hospital admission for heart failure as end-points, were used to illustrate comparisons between quintile groups. Statistical significance was determined using log-rank comparisons. Plasma betaine and *N,N*-dimethylglycine concentrations were adjusted for gender (the mean male betaine concentration was 20.5% higher than the female mean; dimethylglycine was 11.7% higher in males).

The primary urine results are reported on a population excluding subjects taking fibrates (n = 390) because fibrates cause large increases in betaine excretion [28].

Multiple linear regression was used to determine whether plasma betaine, homocysteine or urine betaine excretion were independently associated with an established marker of cardiac injury, plasma NT-proBNP concentration. NT-proBNP and plasma homocysteine levels were log transformed prior to analyses. Cox proportional hazards model regression was used to determine the independence of prognostic markers along with baseline left ventricular ejection fraction, age and gender. Because of evidence that both high and low results were associated with increased events, high (top quintile) and low (bottom quintile) were compared with the middle 60% as categorical variables.

## Results

### Study population

The study population (**Table 1**) was predominantly (72%) male. The standard prescribed medications for coronary patients were present in most cases.

### Association with events

Plasma and urine betaine and betaine metabolites were significantly related to time to event (**Table 2**). Both high and low urinary excretions of betaine were significantly associated with admission for heart failure. The results remained significant when subjects treated with fibrates were included. Low plasma betaine concentrations were associated with acute MIs while high plasma betaine concentrations were associated with admission for heart failure. High plasma concentrations of *N,N*-dimethylglycine and homocysteine were significantly associated with all events, but we did not demonstrate any associations for low levels of either.

**Table 2:** Predictors of events.

	Outcome; mean survival, days (p – value)		
Predictor	Death	AMI	HF
Plasma betaine middle quintiles	1390	1332	1373
Plasma betaine high (>60.6 µmol/L)	1434 (0.7)	1294 (0.18)	**1321 (0.043)**
Plasma betaine low (<33.8 µmol/L)	1354 (0.6)	**1198 (0.014)**	1336 (0.5)
Plasma DMG middle quintiles	1441	1252	1379
Plasma DMG high (>5.8 µmol/L)	**1270 (<0.001)**	**1155 (0.004)**	**1245 (0.027)**
Plasma DMG low (<2.5 µmol/L)	1340 (0.3)	+0.4 (0.5)	1377 (0.12)
Urine betaine excr. middle quintiles	1406	1349	1444
Urine betaine excr. high (>19.5)	1390 (0.5)	1283 (0.6)	**1276 (0.005)**
Urine betaine excr. low (<4.6)	1425 (0.055)	1301 (0.9)	**1291 (0.013)**
Plasma homocysteine middle quintiles	1441	1380	1434
Plasma homocysteine high (>17.0 µmol/L)	**1215 (<0.001)**	**930 (<0.001)**	**987 (<0.001)**
Plasma homocysteine low (<10.0 µmol/L)	1459 (0.13)	1390 (0.6)	1459 (0.092)

Comparisons of the middle three quintiles with the top quintile (“high”) and lowest quintile (“low”) for possible predictors of events. Mean survival times to events given in days, with *p* values (in brackets) for the difference in risk compared with the middle 60% of the population. Significant (p<0.05) statistics are in bold. DMG: *N,N*-dimethylglycine. AMI: acute MI. HF: hospital admission for heart failure. Betaine excretions expressed as mmol betaine/mole creatinine. Plasma betaine and DMG concentrations are gender corrected; male values cited (female values 17% lower for betaine, 10% lower for DMG).

### Details in quintiles of plasma betaine and homocysteine concentrations

**Figure 2:**
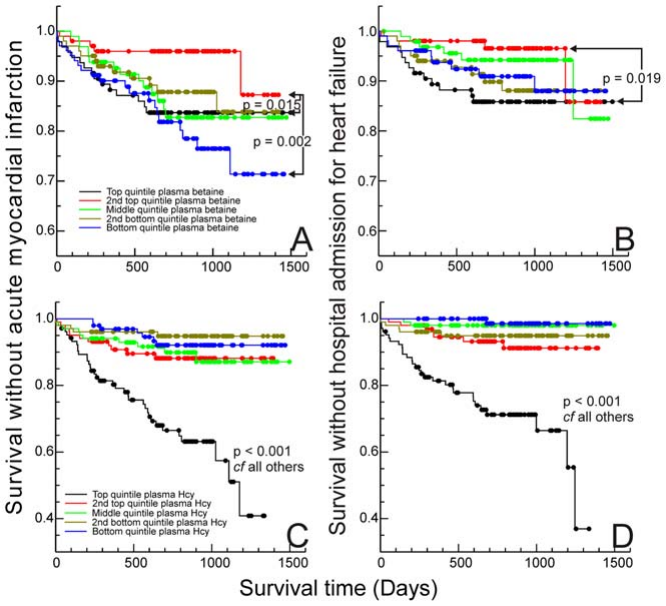
Outcomes in quintiles. Kaplan-Meier curves for time to first secondary event after samples taken: A & B: quintiles of gender-adjusted plasma betaine concentrations, A without acute MI, B without admission to hospital for heart failure; C & D: quintiles of plasma homocysteine concentrations, C without acute MI, D without admission to hospital for heart failure. Significance based on log-rank statistic for paired comparisons.

The prognostic significance of plasma betaine and homocysteine was further examined by comparing outcome in all quintiles of each (**Figure 2**). For all events, the best outcome was for subjects in the second highest quintile of plasma betaine (plasma betaine concentrations 50–60 µmol/L for males, 41–50 µmol/L for females). Compared with this quintile, subjects in both the top quintile, *p* = 0.015, of plasma betaine and (especially) the bottom quintile (<34 µmol/L males, <28 µmol/L females), *p* = 0.002, had an increased risk of secondary acute MI. Only the top quintile showed a significantly increased risk of admission with heart failure (*p* = 0.019). Early events tended to be associated with the top quintile of plasma betaine concentrations (**Figure 2 A&B**).

Only subjects with homocysteine concentrations in the top quintile (>17 µmol/L) of this population had significantly poorer outcomes (**Figure 2 C&D**). Even subjects in the second top homocysteine quintile (13–17 µmol/L), normally regarded as having elevated homocysteine, were not at significantly greater risk. In fact, the increased risk was mainly associated with the top 10% (plasma homocysteine >20.5 µmol/L).

**Figure 3:**
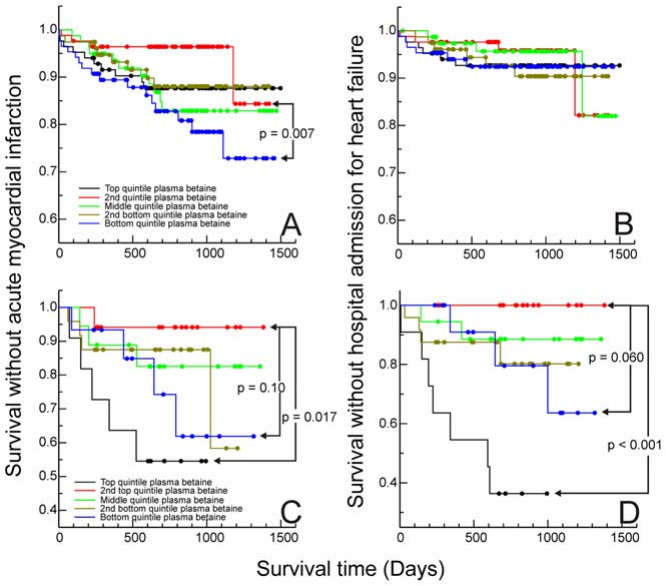
Effect of diabetes. Kaplan-Meier curves for time to first secondary events after samples taken: A & B subjects without diabetes, A time without acute MI, B time without admission to hospital with heart failure; C & D subjects with diabetes, C time without acute MI, D time without admission to hospital with heart failure. Quintiles (based on whole population) of gender-adjusted plasma betaine concentrations. Significance based on log-rank statistic for paired comparisons.

Diabetes frequently alters betaine excretion. In the subjects without diabetes (not taking fibrates, n = 325), only high urinary betaine excretion was confirmed as a risk factor for heart failure (*p* = 0.010). The number of subjects with diabetes (n = 64) was too small for conclusive results, but the trend (*p* = 0.19) was for low, rather than high, betaine excretions to be associated with poor outcomes (admissions for heart failure). There were clearer differences between the groups in the relationship between plasma betaine and MI (**Figure 3 A&C**): in subjects without diabetes (n = 410), patients with low plasma betaine were more likely to have an event (*p* = 0.007) whereas in the patients with diabetes those with elevated plasma betaine had a significantly greater risk (*p* = 0.017). However, given the small sample size, the risk associated with low plasma betaine may be similar in this group (*p* = 0.102) to that in the patients without diabetes. In the subjects with diabetes, plasma betaine was a significant predictor for admission for heart failure (**Figure 3 B&D**), with the top quintile having a higher risk than the second-top quintile (*p*<0.001). Again, given the small numbers, it is possible that heart failure may also be associated with low plasma betaine (for the difference between the second top and bottom quintiles *p* = 0.060).

The plasma betaine quintiles defined groups with similar concentrations of plasma creatinine and plasma urea, and age (**Table 3**). The quintiles differ in median BMI and non-HDL cholesterol, consistent with previous reports [7], [8] that elevations in these are associated with low plasma betaine. Plasma NT-proBNP was also different between the quintiles of plasma betaine, with the lowest concentrations in the middle quintile (**Table 3**).

**Table 3:** Differences between plasma betaine quintiles.

	*p*	Q1	Q2	Q3	Q4	Q5
Pl betaine (µmol/L)	..	28.9	37.6	45.3	54.2	70.0
Age (years)	0.46	66	68	68	68	70
**BMI**	**0.002**	**28.0**	**27.2**	**26.4**	**26.5**	**25.3**
Left ventricular ejection fraction	0.14	60	58	60	60	57
Pl creatinine (µmol/L)	0.31	99	90	100	92	90
Pl urea (mmol/L)	0.28	6.5	5.9	6.5	6.7	6.5
Pl homocysteine (µmol/L)	0.12	13.5	12.3	12.6	12.5	12.3
**Pl Non-HDL cholesterol (mmol/L)**	**<0.001**	**3.38**	**2.98**	**3.22**	**2.97**	**2.64**
**Pl NT-proBNP (pmol/L)**	**0.002**	**71**	**46**	**45**	**71**	**101**
With diabetes (%)	0.18	15	24	18	17	11

Median data in subsets of study population based on quintiles of gender-adjusted plasma (pl) betaine concentrations from Q1 (lowest quintile) to Q5 (highest quintile), with significance (*p*) for difference between quintiles (Kruskal-Wallis one way analysis of variance on ranks). Significant (*p*<0.05) differences between quintiles marked in bold. “With diabetes” row shows percentage of subjects in each plasma betaine quintile who had diabetes.

### NT-proBNP, betaine and homocysteine

**Figure 4:**
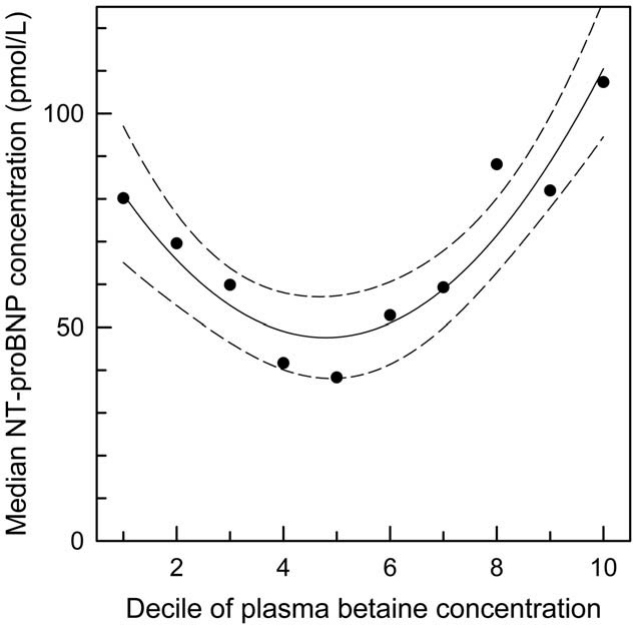
NT-proBNP and plasma betaine. Median NT-proBNP concentrations of deciles of gender-corrected plasma betaine concentrations; trend (quadratic regression) and 95% confidence intervals shown (*p*<0.001).

The trend for NT-proBNP to be elevated either when plasma betaine is low or is elevated (**Table 3**) was confirmed (*p*<0.001) by further dividing the population into deciles of plasma betaine concentration. There was a clear minimum median NT-proBNP in the middle deciles, with both the lowest and top deciles of plasma betaine having higher median NT-proBNP (**Figure 4**).

In a multiple regression model that included age and the presence of renal disease as factors, the log of plasma homocysteine concentration (*p*<0.001) and plasma betaine concentration (*p* = 0.007) were significantly associated with the log of the NT-proBNP concentration. Both factors were positive predictors, and independent (variance inflation factors <1.5), with overall *r* ^2^ = 0.50. Age was also an independent significant predictor (*p*<0.001) but the presence of renal disease was not significant.

### Homocysteine and betaine excretion

Subjects with elevated plasma homocysteine tended to have either high or low betaine excretions, irrespective of whether subjects treated with fibrates were included. Patients with either high (top quintile) or low (bottom quintile) betaine excretion were considered to have “unusual betaine excretion”. In subjects not taking fibrates, 64% of the high homocysteine (>20 µmol/L) subjects had an unusual betaine excretion compared with 39% in the subjects with plasma homocysteine <20 µmol/L (*p* = 0.017). If subjects taking fibrates were included in the comparison the same pattern was found (*p* = 0.005).

### Baseline left ventricular ejection fraction, plasma and urine betaine and age

Cox proportional hazards regression models with age and baseline left ventricular ejection fraction as continuous variables, and gender and betaine groups (Table 2) as categorical variables, were estimated. The betaine groups, highest and lowest quintiles of betaine excretion (patients taking fibrate excluded), were compared with the middle 60%, and the highest and lowest quintiles of gender-corrected plasma betaine were compared with the middle group. Similar models were calculated with plasma homocysteine and plasma *N,N*-dimethylglycine groups. Most of the potential prognostic markers identified in Table 2 were either significant contributors to these models (Table 4) or showed a trend (*p*<0.1) that would justify further investigation.

**Table 4:** Cox regression models.

Prognostic marker *(for outcome)*	Outcome	Hazard ratio (CI)	*p*-value
Top quintile plasma betaine	HF	1.4 (0.7–3.0)	0.30
Top quintile plasma betaine	MI	1.2 (0.6–2.2)	0.64
Top quintile plasma betaine	Death	0.5 (0.2–1.5)	0.22
Bottom quintile plasma betaine	HF	1.2 (0.5–2.9)	0.63
Bottom quintile plasma betaine	MI	**1.9 (1.1–3.5)**	**0.034**
Bottom quintile plasma betaine	Death	0.9 (0.4–2.3)	0.90
Top quintile urine betaine excretion	HF	**2.7 (1.05–2.7)**	**0.039**
Top quintile urine betaine excretion	MI	1.0 (0.5–2.2)	0.99
Top quintile urine betaine excretion	Death	0.6 (0.2–1.8)	0.37
Bottom quintile betaine excretion	HF	**2.7 (1.05–6.9)**	**0.046**
Bottom quintile betaine excretion	MI	1.0 (0.4–2.3)	0.93
Bottom quintile betaine excretion	Death	0.2 (0.02–1.2)	0.077
Top quintile plasma DMG	HF	1.6 (0.8–3.1)	0.20
Top quintile plasma DMG	MI	1.7 (0.9–3.2)	0.076
Top quintile plasma DMG	Death	1.7 (0.5–3.4)	0.10
Top quintile plasma homocysteine	HF	**3.0 (1.4–6.2)**	**<0.001**
Top quintile plasma homocysteine	MI	**2.6 (1.4–4.7)**	**0.002**
Top quintile plasma homocysteine	Death	2.1 (0.9–4.8)	0.082

Cox proportional hazards regression models with admission for heart failure (HF, acute myocardial infarction (MI) or death (all causes) as the outcomes. Models included left ventricular ejection fraction (LVEF) and age as continuous variables. Categorical variables were gender, and high, middle or low plasma betaine concentration or urine betaine excretion (Table 2), or plasma homocysteine or *N,N*-dimethylglycine (DMG): the middle 60% group used as the reference group. Significant (p<0.05) markers in bold. DMG: *N,N*-dimethylglycine. CI: 95% confidence interval.

## Discussion

### Betaine metabolism and coronary artery disease

Our results are direct evidence that both high and low plasma betaine concentrations are associated with an increased coronary risk, a common pattern with essential homeostatically controlled metabolites. These observational results do not establish causality, and were obtained on a selected population with a pre-existing high risk of further events. Betaine (both plasma concentrations and excretion) is clearly a marker of this risk; betaine excretion is associated with an increased risk of heart failure rather than MI. High excretion may persist for years [29] and may cause a betaine insufficiency, while a primary betaine insufficiency is a likely reason for an abnormally low excretion. These results support inferences from cross-sectional data [6], which suggested that there could be an association between betaine excretion and vascular disease. Increasing betaine metabolism is a major response to an increased supply of homocysteine (**Figure 1**), and this results in an elevation in plasma *N,N*-dimethylglycine, which is also associated with more events. Low plasma betaine has previously been associated with an unfavorable risk profile in a general adult population [7], particularly in the metabolic syndrome, and a similar significant association of low plasma betaine with an unfavorable lipid profile and high BMI was also found in the present cohort [8]. Another cross-sectional study associated high plasma betaine with an increased risk of vascular disease [15], and the present study suggests that these reports are consistent. Our results could also help to clarify the “homocysteine controversy” [30]. Homocysteine has been reaffirmed as a strong risk factor for, or marker of, cardiovascular disease [31], [32] but using folate supplementation to lower plasma homocysteine concentrations does not lead to a decrease in the incidence of secondary events [17]–[19]. In the present cohort, homocysteine is confirmed as a powerful risk factor. This study does not have the power to show whether modest elevations of plasma homocysteine show a graded risk increase, but the relationship between plasma homocysteine and risk is clearly non-linear, and the difference between the reported mean plasma homocysteine concentrations of the folate-treated and control groups in the larger studies would not be associated with a significant difference in risk in our cohort. The high homocysteine concentrations that we have found to be associated with an increased risk were also associated with disturbed betaine metabolism, and the possibility that the larger study populations included subjects with a betaine insufficiency appears to have been overlooked.

We found that homocysteine and plasma betaine are independent predictors of plasma NT-proBNP; the anomaly that plasma betaine is also positively associated with NT-proBNP is consistent with the negative association between the natriuretic peptide and obesity [33], [34], since plasma betaine is also negatively associated with obesity [7]. The association of NT-proBNP with betaine is stronger than with BMI or blood lipids and is confounded by a trend for NT-proBNP to also increase when plasma betaine is low (**Figure 4**), an observation that supports the conclusion that both low and elevated plasma betaine are associated with pathological changes. A recent cross-sectional study has suggested that the association between NT-proBNP and homocysteine is linked through impaired fatty acid oxidation [35]. While it is clear that plasma betaine, betaine metabolism and betaine excretion are markers of outcome, it has not been established that they are independent of other markers, or that betaine has a mechanistic role in the pathology. There were too few events in this study to establish the increase in risk associated with markers of betaine metabolism (as shown by the wide confidence intervals in Table 4), but the preliminary evidence suggests that these possibilities should be investigated.

### Betaine insufficiency

Betaine is essential for cell volume regulation [1]–[4] and most tissues contain millimolar concentrations of intracellular betaine [9], much higher than circulating concentrations. This tissue betaine (**Figure 1**) is also a major store of methyl groups for the methylation processes that are essential for diverse functions, including creatine phosphate and phospholipid biosynthesis and the epigenetic control of gene expression. Therefore a betaine insufficiency may itself be pathogenic. Betaine insufficiency may be common in the metabolic syndrome [3], [7], and betaine deficient subjects may be at greater risk of cardiovascular events. This hypothesis is difficult to test because plasma betaine is regulated and does not appear to change with tissue betaine, for example, in diuresis and antidiuresis [12]. Plasma betaine is a poor measure of tissue betaine [9]. Some subjects, especially those with diabetes mellitus [3] lose excessive amounts of betaine and these indeed do have a significantly increased risk of heart failure. In subjects with diabetes high betaine excretion is associated with the loss of another renal osmolyte, sorbitol [36]. Betaine loss may persist for years [29], and the amount lost is often comparable with the normal daily betaine intake. Such subjects are candidates for a secondary betaine insufficiency. Normal betaine excretion probably reflects loss from the renal medulla during osmoregulation [37] rather than incomplete tubular resorption because it does not correlate with plasma betaine [3], is low (the fractional clearance is less than 2%) but approximately constant for individual subjects [12], [14], and is minimally affected by either osmotic status [13] or by betaine intake [10], [11]. Unusually low betaine excretions may reflect a primary betaine deficiency, which could be a result of poor diet or impaired mitochondrial oxidation of choline. This is speculative, but it would explain why low betaine excretion also appears to be associated with secondary cardiovascular events and with elevated plasma homocysteine in the present population.

The possibility that high plasma betaine concentrations may also be a risk factor [15] probably reflects a different pathology, for example, a dysfunction in the control of betaine efflux from tissues, where betaine concentrations are much higher than in blood [9]. Efflux is normally tightly regulated [38], [39], so a high blood concentration could be associated with a tissue insufficiency. Alternatively, high plasma betaine could result from decreased betaine homocysteine methyltransferase activity, as has been observed in animal models [40], [41]. In our study a minority of subjects who had events had high plasma betaine concentrations, and these subjects tended to have early events; this was especially evident in patients with diabetes, consistent with significant tissue damage at baseline. The contrast between patients with and without diabetes points to differences in the sample populations as an explanation for the contradictions between cross-sectional studies where low plasma betaine is associated with unfavourable vascular risk profiles [7], [8] and those where high plasma betaine appears as a risk factor [15].

### Betaine insufficiency and homocysteine

The most satisfactory test for betaine status, the methionine load test [2], [3], [16], is not appropriate for screening seriously ill subjects. Fasting homocysteine is also affected by the availability of betaine [3], [16], a result of the action of the liver and renal enzyme betaine-homocysteine methyltransferase which catalyzes the remethylation of homocysteine to methionine, the other product being *N,N*-dimethylglycine (**Figure 1**). The importance of this pathway has been demonstrated by blocking [40] or deleting [41] this enzyme, leading to a large increase in plasma homocysteine with a simultaneous increase in plasma betaine. Since this is the only known metabolic source of dimethylglycine, an increase in plasma dimethylglycine indicates that betaine is being consumed, though its rapid clearance means that this may not be a sensitive test with fasting samples. Fasting plasma homocysteine is well-known to be affected by vitamin intake (particularly folate and B_12_) as well as by the betaine supply, making this a nonspecific test.

The results reported here confirm that elevated homocysteine is a strong risk factor for later events in people with established coronary artery disease, though the overall result is mainly attributable to a small number of subjects with exceptionally elevated (>20 µmol/L) plasma homocysteine concentrations. A number of intervention studies in recent years have shown that lowering plasma homocysteine in large populations by supplying folate and other B-vitamins does not decrease the incidence of secondary vascular events [17]–[19]. This anomaly could be resolved if some of the cases of elevated homocysteine in subjects with vascular disease reflect betaine insufficiency, which is plausible because the metabolic syndrome is likely to be common in these subjects. Folate supplementation will not correct a betaine insufficiency. It lowers homocysteine in most subjects, but the reduction in risk is relatively small in subjects with normal or only mildly elevated homocysteine concentrations, compared with the large risk associated with plasma homocysteine >20 µmol/L. A majority of the examples of highly elevated plasma homocysteine appear to be associated with evidence of a betaine insufficiency, and the subjects with both high homocysteine and possible betaine insufficiency are at greater risk of secondary events, especially heart failure.

### Betaine sufficiency and blood lipids

Given that betaine is highly water soluble and lipophobic, it is surprising that the supply of betaine affects lipid metabolism, with the varied explanations unconvincing [3], [5]. Human cross-sectional studies have suggested that that these relationships are relevant in human populations [7] including the present one [8], and that low plasma betaine concentration is associated with the metabolic syndrome and known prognostic markers of vascular disease. In subjects with elevated blood lipids, betaine loss in the urine is associated with elevated plasma homocysteine [42], [43]. These relationships have been discussed elsewhere for the present population [8], [43]. Lipid lowering drugs do have an effect: fibrates appear to strongly affect the urinary excretion of betaine [28] and therefore subjects taking fibrates were excluded when the urinary results were analyzed here. Statin therapy is associated with a mild increase in plasma betaine concentrations [8], [43] but the difference is small compared with the gender difference. Other cardiovascular drugs also may affect betaine handling [43]. Given that most of the evidence is cross-sectional, it is not at all clear whether betaine deficiency causes dyslipidemia, is a consequence of dyslipidemia, or both are markers of an underlying pathology, and this needs to be clarified.

### Should betaine insufficiency be corrected?

Our results are consistent with the hypothesis that a tissue betaine insufficiency will increase the incidence of secondary heart failure. Impaired osmoregulation may be an important mechanism. Betaine insufficiency may also increase the incidence of secondary MI, but the evidence is less persuasive, since low plasma betaine concentrations and increased metabolism to dimethylglycine could be the result rather than the cause of disease. Possibly the role of betaine as a methyl store may be more significant for risk of MI. These inferences are speculative on the basis of our observational evidence, but they suggest that interventional studies are justified.

Betaine supplementation is inexpensive and safe [1], [3], [44]. It has been used in the animal industries to decrease the fat content, and increase the lean muscle mass, of meat [5]; it improves the atherogenic risk factor profile of a mouse model [45], and in an apolipoprotein E-deficient mouse model attenuates atherosclerotic lesions [46]. It has recently been used to enhance human athletic performance measures such as power and endurance [47]–[49]. Therefore, in the absence of a simple test for betaine sufficiency, the hypotheses suggested here could be best be addressed directly, initially by some pilot randomized controlled trials of betaine supplementation using appropriately selected at-risk populations.

### Limitations

The main limitations of this study are inherent in the chosen population: this clinical cohort is not representative of the general population. Because the subjects have existing disease, there is ambiguity about the extent to which the markers are detecting base line disease or are prognostic of the development of new disease. The small number of subjects with diabetes limits the power to detect effects in this sub-group and negative results on these patients are inconclusive. The observational results do not provide insights into mechanisms and can only generate hypotheses.

### Conclusions

Disturbances in betaine homeostasis are associated with secondary events in an acute coronary syndrome cohort. Betaine insufficiency should be considered as a possible cause of elevated homocysteine. The possibility that correcting betaine insufficiency could improve outcomes should be investigated.
